# Outbreak of Infections Caused by *Shigella sonnei* with Decreased Susceptibility to Azithromycin — Los Angeles, California, 2012

**Published:** 2013-03-08

**Authors:** Roshan Reporter, Marifi Pulido, Anna Bowen, Maria Sjolund Karlsson

**Affiliations:** Acute Communicable Disease Control Unit, Los Angeles County Dept of Public Health; Div of Foodborne, Waterborne, and Environmental Diseases, National Center for Emerging and Zoonotic Infectious Diseases, CDC

In May 2012, the Los Angeles County Department of Public Health’s Acute Communicable Disease Control Unit and Environmental Health, Food, and Milk Program investigated an outbreak of shigellosis associated with a private bridge club. This investigation documented the first known transmission of *Shigella sonnei* with decreased susceptibility to azithromycin in the United States.

Cases were defined as an illness clinically compatible with shigellosis in a patient or *S. sonnei* isolated from stool of a person with an epidemiologic link to the bridge club during May 22–26, 2012. Investigators attempted to interview all bridge club workers and members who had visited the bridge club during the week of May 22; they collected stool specimens from workers who handled food and from workers and members with diarrhea who had not already submitted a stool specimen for culture at a health facility. Thirty-nine cases were identified among club members with diarrhea and four among club workers; of the four workers, two, including one who handled food, reported no symptoms. The average age of affected persons was 75.3 years (range: 54–98 years); 55% were female. Among those with symptoms, the duration of illness averaged 5.9 days (range: 1–14 days). Common symptoms included diarrhea in 95% of patients, abdominal cramps in 70%, and fever in 56%. Thirty-one (72%) persons sought medical care, and 10 (23%) were hospitalized. No specific exposures implicated a source for the outbreak.

Among the 43 cases, 14 were culture-confirmed; 10 isolates underwent pulsed-field gel electrophoresis (PFGE), yielding indistinguishable patterns. Four isolates submitted to CDC’s National Antimicrobial Resistance Monitoring System (NARMS) displayed resistance to streptomycin, sulfisoxazole, tetracycline, and trimethoprim-sulfamethoxazole. Unlike most *Shigella* isolates tested by NARMS, these isolates also showed elevated azithromycin minimum inhibitory concentrations (MICs) of >16 *μ*g/mL (1) and harbored a plasmid-encoded macrolide resistance gene, *mphA* (2).

CDC’s PulseNet identified two additional isolates indistinguishable from the outbreak PFGE pattern. One was from a man in Pennsylvania aged 23 years who had visited Los Angeles in April, and the other from a man in Hawaii aged 53 years who visited Los Angeles during April and May; both men were hospitalized with diarrhea. Neither case was epidemiologically linked to the bridge club or to each other.

Although sporadic cases of shigellosis caused by *Shigella* strains with increased azithromycin MICs have occurred, this is the first outbreak documented in the United States and might indicate increasing circulation of such strains (1). Illnesses in this outbreak tended to be severe; however, the affected population was much older than the general U.S. population. Clinical management of such illnesses is likely to be complex; although azithromycin currently is recommended for treatment of infections caused by multidrug-resistant *Shigella,* options for alternative treatment among children with such infections primarily include parenteral antimicrobial medications (3,4).

Guidelines for azithromycin susceptibility testing and criteria for interpretation of MICs for *Shigella* species have not been published. Clinicians are urged to report azithromycin treatment failure among shigellosis patients to public health authorities and to retain *Shigella* isolates from such cases for further analysis.
